# Analysis of the construct of dignity and content validity of the patient dignity inventory

**DOI:** 10.1186/1477-7525-9-45

**Published:** 2011-06-19

**Authors:** Gwenda Albers, H Roeline W Pasman, Mette L Rurup, Henrica CW de Vet, Bregje D Onwuteaka-Philipsen

**Affiliations:** 1 Department of Public and Occupational Health and the EMGO Institute for Health and Care Research; VU University Medical Center, Amsterdam, Van de Boechorststraat 7, 1081BT, The Netherlands; 2 Department of Epidemiology and Biostatistics and the EMGO Institute for Health and Care Research, VU University Medical Center, Amsterdam, Van de Boechorststraat 7, 1081BT, The Netherlands

## Abstract

**Background:**

Maintaining dignity, the quality of being worthy of esteem or respect, is considered as a goal of palliative care. The aim of this study was to analyse the construct of personal dignity and to assess the content validity of the Patient Dignity Inventory (PDI) in people with an advance directive in the Netherlands.

**Methods:**

Data were collected within the framework of an advance directives cohort study. This cohort study is aiming to get a better insight into how decisions are made at the end of life with regard to advance directives in the Netherlands. One half of the cohort (n = 2404) received an open-ended question concerning factors relevant to dignity. Content labels were assigned to issues mentioned in the responses to the open-ended question. The other half of the cohort (n = 2537) received a written questionnaire including the PDI. The relevance and comprehensiveness of the PDI items were assessed with the COSMIN checklist ('COnsensus-based Standards for the selection of health status Measurement INstruments').

**Results:**

The majority of the PDI items were found to be relevant for the construct to be measured, the study population, and the purpose of the study but the items were not completely comprehensive. The responses to the open-ended question indicated that communication and care-related aspects were also important for dignity.

**Conclusions:**

This study demonstrated that the PDI items were relevant for people with an advance directive in the Netherlands. The comprehensiveness of the items can be improved by including items concerning communication and care.

## Introduction

Dignity is a topic which often arises in discussions about care for dying patients. Since the concept of dignity is not clearly defined in palliative care, the term dignity is used in many different ways, and easily evokes confusion. Although, several authors have argued that dignity should be considered as a central principle in palliative care [1-3], and that conserving dignity can be considered as a goal of the care that is provided [4-7].

Dignity can be defined as the quality of being worthy of esteem or respect. A distinction can be made between two types of dignity: basic dignity and personal dignity. Basic dignity is the inherent dignity of every human being, which nothing can take away, and personal dignity refers to a personal sense of worth, associated with personal goals and social circumstances. It is related to a persons' self-esteem and perceptions of being respected by others, and consequently it can be taken away or enhanced [8,9]. The current study focused on personal dignity at the end of life.

Preserving dignity is frequently mentioned by patients when considering the end of life. Consequently, concern about loss of dignity is one of the most common reasons why people formulate an advance directive in the Netherlands [10]. In addition, loss of dignity is one of the most frequently mentioned reasons for requesting euthanasia or physician-assisted suicide [11,12]. The law in Oregon concerning physician-assisted suicide is called 'the Oregon Death with Dignity Act' [11]. Hence, considering end-of-life care from patient perspective the concept of personal dignity can contribute to palliative care research.

An adequate measurement instrument to identify aspects that cause distress at the end of life will provide insight into the issues that are relevant and important for a person's sense of dignity. Understanding the causes of dignity-related distress could help to improve palliative care and research in palliative care.

Based on a qualitative study focusing on how dying cancer patients in Canada understand and define dignity, Chochinov et al. developed an empirical model of dignity to understand how patients face an advancing terminal illness [13]. Items were developed from the themes and sub-themes in the model, and terminally ill cancer patients were asked how much they thought that these items could influence their sense of dignity. In this way the dignity model was validated, and a first draft of the Patient Dignity Inventory (PDI) was developed [14]. This 22-item PDI prototype was later revised and became the 25-item PDI, a measurement instrument which can be used by clinicians to detect end-of-life dignity-related distress [15].

In Canada the PDI has been found to be a valid and adequate instrument for use in patients with terminal cancer, but it is unclear if and to what extent the PDI items are relevant for other groups of patients or for patients in other countries. Some people, when they get older, or they or their loved ones have been confronted with disease, become concerned about their dignity, think about their wishes with regard to end-of-life care, and formulate an advance directive.

Advance directives are documents in which one can state one's preferences concerning end-of-life care, aimed at making someone's wishes known in situations where he/she is not able to do so in another manner. In the Netherlands, the most common standard advance directives, the advance euthanasia directive, the refusal of treatment statement and the durable power of attorney (appointment of a health care representative) are provided by the Right to Die-NL, and the wish to live statement (stating the wish to receive adequate care directed at quality of life, and explicitly refusing euthanasia), is provided by the Dutch Patient Association.

Given that people with an advance directive have thought about and realise the importance of end-of-life issues, it is of great interest to study their ideas about dignity, because these can be very useful for health care providers in organising advance care-planning. Therefore, we performed a content analysis of the construct of personal dignity for a broader population than cancer patients, to investigate which items influence personal dignity for people with an advance directive in the Netherlands. Furthermore, we investigated the content validity of the PDI by assessing the relevance and the comprehensiveness of the PDI items with the COSMIN checklist (COnsensus-based Standards for the selection of health status Measurement INstruments) [16,17].

## Methods

### Design and study population

**Figure 1:**
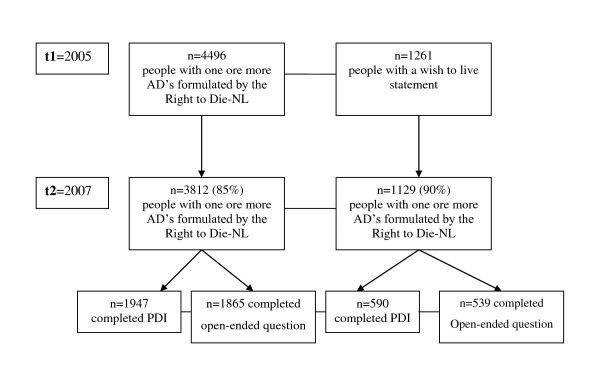
**Flow chart of recruitment and response rates**.

The data for this study were collected within the framework of the Advance Directives Cohort Study [18]. The study was approved by the Medical Ethics Review Committee of the VU University Medical Center. The Advance Directives Cohort Study is a major ongoing longitudinal study aiming to get insight into how advance directives are involved in end-of-life decisions in the Netherlands. This cohort study started in 2005, and follow-up measurements are performed once every one and a half years. The design of the Advance Directives Cohort is described in detail by Van Wijmen et al.[18]. The data used in the present study were collected during the second cycle of data collection. A written questionnaire with structured questions was sent to the cohort of participants with one or more of the most common standard advance directives in the Netherlands provided by the Right to Die-NL and the Dutch Patient Association. During the first data-collection cycle the cohort consisted of 4,496 people who had one or more advance directives formulated by the Right to Die-NL, and 1,261 people who had a wish to live statement. The response rate in the second data-collection cycle was 85% respectively 90% for the Right to Die-NL members and the members of the Dutch Patients Association (see Figure 1).

The present study is based on data which were collected in the Spring of 2007. We randomly split the cohort into two by alternately placing cases in one of two subsamples; one half received a questionnaire which included an open-ended question concerning important factors for personal dignity, and the other half received the PDI. Accordingly, there were four groups: 1) people with one or more advance directives from the Right to Die-NL who received the open-ended question, 2) people with one or more advance directives from the Right to Die-NL who received the PDI, and 3) people with a wish to live statement who received the open-ended question, and 4) people with a wish to live statement who received the PDI. A total of 3,812 people with one or more advance directives (95% had an advance euthanasia directive, 65% had the refusal of treatment statement, and 63% had the durable power of attorney) and 1,129 members of the Dutch Patient Association completed the questionnaire in the second data-collection cycle.

### Measurement instrument

All respondents were asked some questions about demographic characteristics and how they rated their health status (very good; good; less than good).

As described above, one randomly selected half of the cohort received an open-ended question, which was introduced with the following text: 'The term dignity is often used when talking about the last phase of life. However, little is known about what exactly influences a person's sense of dignity'. These respondents were asked two questions: 'Please describe how you would define dignity", and 'what issues do you think that would influence your sense of dignity during the last phase of their life?'.

The other randomly selected half of the cohort received the PDI, in which they were asked to rate the extent to which they though the items could influence their sense of dignity during the last phase of life, on a 5-point scale (1 = not at all; 2 = slightly; 3 = moderately; 4 = a lot; 5 = very much). The PDI was introduced with a text similar to that introducing the open-ended question. In order to assess the comprehensiveness of the PDI items, the respondents were also asked whether they thought that there were any items missing in the PDI which could influence their sense of dignity during the last phase of life.

This study is based on the PDI prototype, a measurement instrument that can be used to assess various sources of dignity-related distress among cancer patients nearing the end of life [14]. This first version of the PDI consists of 22 items, divided into four domains (i.e. psychological, physical, social and existential) that influence the sense of dignity of terminally ill cancer patients. The items were translated into Dutch by means of forward and backward translation. The PDI items were independently translated from English to Dutch by two researchers. Two other researchers with no knowledge of the PDI of whom one native speaker did the backward translation. The two backward translations were compared and only small differences were found and resolved by consensus. Subsequently, the Dutch version was tested in a pilot study consisting of people with an advance directive. The pilot showed that the item "Thinking how life might end" was not considered as influential to sense of dignity at the end of life. This might have been expected since the majority of the study population was in good health. Therefore, we decided to exclude this item of the original PDI prototype.

### Analyses

We analysed the responses to the open-ended question to address the first aim of this study, i.e. the content analyses of the construct of dignity. We first organised the data obtained from the responses to the open-ended question. Sub-themes referring to any aspect of dignity were assigned to all of these responses and content labels were assigned to the sub-themes. We started off by structuring our labels according to the four domains (physical, psychological, social, existential) and the PDI items distinguished by Chochinov et al. These domains were used as layers for the four columns within a scheme in which the content labels were placed. Two researchers (familiar with the PDI) independently read and applied content labels to 400 responses open-end responses. These labels were compared, and any disagreements between the researchers were discussed and resolved. This process continued until there was complete consensus regarding the labelling, and no additional content labels were assigned or added to the scheme.

The COSMIN checklist was used to address the second aim of this study, which was to analyse the content validity of the PDI. According to the COSMIN taxonomy of measurement properties, which is based on an international Delphi study, content validity is defined as: the degree to which the content of a measurement instrument is an adequate reflection of the construct to be measured [19]. As described above, in this study the construct of dignity was defined by the issues that were mentioned as important for dignity in the responses to the open-ended question. According to the COSMIN checklist, 5 questions should be answered to assess content validity (Table 1).

**Table 1:** Content validity box from the COSMIN checklist

Box D. Content validity (including face validity)				
*General requirements*		yes	no	?
1	Was assessed if all items refer to relevant aspects of the construct to be measured?	□	□	□
2	Was assessed if all items are relevant for the study population? Considering e.g. age, gender, disease characteristics, country, setting	□	□	□
3	Was assessed if all items are relevant for the purpose of the application of the measurement instrument? i.e. (1) discriminative (distinguish between groups at one point in time), (2) evaluative (assess change over time), and/or (3) predictive (predict future values)	□	□	□
4	Was assessed if all items together comprehensively reflect the construct to be measured in terms of (1) content coverage and description of domains, and (2) the theoretical foundation?	□	□	□
5	Were there any important flaws in the design or methods of the study?	□	□	

* The response rates are not corresponding with the response rates in the paper describing the design of the Advance Directives Cohort Study [18] since we excluded people who were only member of the Right to Die-NL and did not complete an advance directive (at that time).

First, we assessed whether all items of the PDI were represented in the responses to the open-ended question *(COSMIN requirement 1)*.

Secondly, we assessed whether the focus and detail of the content of the PDI match the target population. In other words, we assessed whether each PDI item was relevant for the study population by calculating the percentage per item of people who scored 4 or 5 on the 5-point scale. These percentages indicate how many people considered that the items would influence dignity at the end of their life *(COSMIN requirement 2)*. In this way, the study population judged the relevance of the items. In addition, we checked the number of missing observations given that many missing observations on an item can be an indication that the item is not relevant for the population.

The third COSMIN requirement determines whether all items are relevant for the purpose of the application of the instrument. This items is not applicable since this study aims to examine whether the PDI items are relevant for a population different from the population in which the instrument was originally developed. In this study the instrument has not been subjected to a discriminative, evaluative or predictive application.

In addition, we assessed whether the PDI items comprehensively reflect the construct of dignity. Hence, we assessed the extent to which issues mentioned as important for a person's sense of dignity in the responses to the open-ended question were represented in the PDI items *(COSMIN requirement 4)*.

The last COSMIN item *(COSMIN requirement 5) *determines whether there are any important flaws in the design or methods of the study. This item is only applicable when evaluating a study, and not when performing a study to assess the content validity of health measurement instruments.

## Results

### Response rates

The response rate in the people who received the questionnaire including the PDI varied per item, from 88% to 92% among people with an advance directive from the Right to die-NL and from 80% to 84% in people with a wish to live statement. The majority of the people who received the open-ended question could describe how they understand dignity and could also describe some issues which they thought would influence their sense of dignity during the last phase of their life. The response rate was 91% and 82%, respectively, in the people with an advance directive from the Right to die-NL and the people with a wish to live statement who received the open-ended question.

### Characteristics of the respondents

Table 2 presents the characteristics of the respondents. More than half of all the respondents were female, and the mean age in all groups was between 60 and 70 years of age. Almost all people with a wish to live statement had religious beliefs, compared to 36% of the people with an advance directive formulated by the Right to die-NL. The study population consisted of people with different ratings for health status, a majority of whom assessed their health status as good.

**Table 2:** Characteristics of the people with one or more advance directives from the Right to die-NL and people with a wish to live statement

Characteristics	People with an advance directive from the Right to die-NL		People having a wish to live statement	
	**PDI** **n = 1947**	**Open-ended** **question** **n = 1865**	**PDI** **n = 590**	**Open-ended question** **n = 539**

Kind of advance directive				
- Advance euthanasia directive	95	94		
- Refusal of treatment document	65	64		
- Durable power of attorney	63	63		
Sex, female %	61	68	60	59
Age mean (SD) [range]	69 (12) [26-98]	70 (12) [25-100]	61 (17) [17-92]	62 (17) [19-92]
Marital status %				
Single/divorced/widowed	41	42	29	28
Married or with partner	59	58	71	72
Level of education^1 ^%				
Low	5	6	13	16
Intermediate	55	56	66	60
High	40	38	21	24
Religious beliefs %	35	37	99	99
Self perceived health status				
Very good	19	20	22	19
Good	59	58	59	61
Less than good	22	23	16	19

^1 ^Low: Lower vocational education; lower secondary general education; primary school. Intermediate: Intermediate vocational or higher secondary general education. High: Higher vocational education; university.

### Construct of dignity

All issues mentioned in the responses to the open-ended question were used to define the construct of dignity in this study. The Additional file 1, Table S1 contains a list of issues which were considered to influence dignity by people with an advance directive, and which consequently define the content of the construct of dignity. Issues most frequently mentioned were: independence, incontinence, pain, mental clarity, dementia, the ability to communicate and adequate care. During the coding process it became apparent that care-related aspects were not covered by any of the domains, but were thought to influence dignity, so we added care as a sub-theme.

### Relevance of the PDI items

Analysing the content validity of the PDI, we assessed the relevance of the PDI items for (1) the construct to be measured, (2) the study population, and (3) the purpose of the study.

Firstly, the majority of the PDI items were relevant for the construct to be measured, because they were represented in the responses to the open-ended question. However, some PDI items, i.e. 'changes in physical appearance', 'not being able to carry out important roles', 'not feeling you made a meaning or lasting contribution', 'not being able to mentally fight', 'not being able to accept things the way they are' and 'uncertainty regarding illness' were not or only (very) seldom reflected in the responses to the open-ended question *(COSMIN requirement 1)*. In accordance, these PDI items were the least frequently indicated as influential for dignity by the respondents who completed the PDI *(*see Table 3*)*.

**Table 3:** PDI items considered to influence sense of dignity at the end of life by people with one or more advance directives from the Right to die-NL and people with a wish to live statement

	Range of distribution Mean (SD)	People with an advance directive from the Right to die-NL n = 1947%*	People with a wish to live statement n = 590%*
*Physical aspects*			
Not being able to independently manage bodily functions	3.7 (1.3)	73	41
Not being able to carry out tasks of daily living	3.4 (1.3)	58	28
Not being able to continue with usual routines	3.1 (1.2)	45	27
Experiencing distressing symptoms	3.1 (1.1)	37	31
Not being able to carry out important roles	2.7 (1.2)	29	19
Changes in physical appearance	2.2 (1.1)	12	18
			
*Psychological aspects*			
Not being able to think clearly	3.8 (1.2)	73	53
Not being able to mentally fight	3.6 (1.2)	61	38
Feeling depressed or anxious	3.3 (1.2)	51	42
Not being able to accept things the way they are	3.2 (1.3)	45	36
			
*Social aspects*			
Feeling a burden to others	3.8 (1.3)	74	50
Not being treated with respect or understanding	3.4 (1.3)	52	57
Feeling your privacy has been reduced	3.2 (1.2)	49	38
Not feeling supported by your community	3.2 (1.3)	43	48
			
*Existential aspects*			
Feeling you do not have control over your life	3.6 (1.3)	67	38
No longer feeling like who you were	3.5 (1.3)	59	45
Feeling life no longer has meaning or purpose	3.3 (1.4)	58	33
Not feeling worthwhile or valued	3.2 (1.3)	43	44
Not having a meaningful spiritual life	2.9 (1.4)	33	41
Uncertainty regarding illness	2.9 (1.2)	31	33
Not feeling you made a meaning or lasting contribution	2.6 (1.2)	23	21

* Percentage that agree or strongly agree (scored a 4 or 5 on a 5-point scale) that the aspect influence the sense of dignity during the last phase of life

∞21 items are included because the item "Thinking how life might end" of the original PDI prototype was excluded from the current study as a result of a pilot study

Secondly, Table 3 shows the mean and SD together with the percentages of (strong) agreement, indicating that each PDI item is considered to influence dignity at the end of life *(COSMIN requirement 2)*. However, one of the items, 'changes in physical appearance' was only considered to influence sense of dignity by a small number of respondents in both groups, so it might be considered to be less relevant for the present study population.

### Comprehensiveness of the PDI items

Finally, a comparison of the results from the PDI and the responses to the open-ended question *(COSMIN requirement 4*) showed that most issues described in the responses were covered by the PDI items.

Issues not represented in the PDI were aspects related to care and the ability to communicate. Table 4 shows that communication as a way of indicating what a person wants, and communication as a social activity, are both thought to be issues that are relevant for dignity at the end of life. In addition, Table 5 shows a variety of care-related issues which are considered to be important for dignity.

**Table 4:** Content labels applied to responses to the open-ended question concerning social aspects

SOCIAL
Being able to communicate (in general)
Communication as a means of indicating what a person wants
Communication as a social activity

**Table 5:** Content labels applied to responses to the open-ended question concerning care related issues

CARE
***Environmental aspects of care***
Being cared for in a quiet/safe place
Being cared for at home/not in an institution
Not being cared for by strangers/many different people
Being cared for in a hospice
***Desired treatment goals***
No unnecessary prolongation of life/being allowed to 'let go'
(No) hastened death/euthanasia
Adequate pain (and symptom) management/relief of suffering
Relief suffering
Palliative care
***Care characteristics***
Adequate care/tailored care
Warm loving care
Spiritual support

The people who completed the PDI indicated that communication and care-related aspects were issues which were missing in the PDI, as well as the following issues: independence, pain, incontinence, dementia, being treated with respect, and the ability to wash, eat and drink independently, and to go to the toilet without help.

The responses to the open-ended question described the issues in more detail, or in a different way, compared to the PDI items. For example, the PDI item 'not being able to independently manage bodily functions' is represented in the following issues mentioned in the responses to the open-end question, but more specifically described as: incontinence, and being able to wash, eat and drink independently (see Table 6).

**Table 6:** Content labels applied to responses to the open-ended question concerning physical issues

PHYSICAL
Independence
*Not being able to independently manage bodily functions (PDI item)*
*Not being able to carry out tasks of daily living (PDI item)*
Incontinence
Not being able to wash and bath independently
Not being able to eat/drink independently
Immobile/bedridden

## Discussion

With the COSMIN checklist we assessed the content validity of the PDI in people with an advance directive in the Netherlands. All of the PDI items, apart from the item "Thinking how life might end", were thought to be relevant to sense of dignity at the end of life by people with an advance directive formulated by the Right to die-NL, and by people with a wish to live statement. However, the PDI items did not comprehensively reflect the construct of dignity, because the PDI lacks items about communication and care characteristics. In the responses to the open-ended question these were mentioned as important issues that influence dignity and these were also indicated as missing items in the PDI.

### PDI items versus responses to open-ended question

The issues that were most frequently indicated as important for sense of dignity, such as the ability to manage bodily functions, the ability to think clearly and feeling a burden to others, in the responses to the open-ended question also received the highest scores in the PDI, and vice versa PDI items that were the least frequently mentioned as influential for dignity, such as changes in physical appearance were also the issues that were the least frequently mentioned in the responses to the open-ended question, although the latter gave more detailed information.

The respondents who completed the PDI indicated that they missed items in the PDI, for instance about the ability to wash, eat and drink independently, and to go to the toilet without help. Nevertheless, these issues are basically represented by the PDI item 'not being able to independently manage bodily functions'. This indicates that the PDI items are quite abstract, and are not clear for all respondents. People possibly prefer more specific phrasing such as, 'not being able to independently get to the toilet'.

The responses to the open-ended question show that being able to communicate and care-related aspects are relevant for a person's sense of dignity, whereas these issues are not included in the PDI. However, communication and various care-related issues were mentioned as missing items in the PDI, demonstrating once more that these are important issues. In Chochinov's model of dignity, care tenor is recognised as a sub-theme of the social dignity inventory. It relates to the attitudes other people demonstrate when interacting with a patient [13]. Care tenor is represented by the PDI item concerning being treated with respect. However, this item is very general, and does not specify how the attitudes of health care providers influence a person's dignity. The revised 25-item PDI includes an additional item: 'not feeling supported by my health care providers'. In addition, in a study investigating the dignity-conserving model, it was found that staff had a considerable impact on the sense of dignity of people living in nursing homes [20]. Nevertheless, the present study indicates that care-related aspects, e.g. the location of care also influence dignity. Even though the care-related aspects are not covered by the social domain, and required the addition of a separate care domain, and the results of this study demonstrated the importance of care and communication for dignity, it is still debatable whether a separate domain for care is the best option.

### Use of PDI in people with an advance directive

The respondents were asked what issues they thought would influence their sense of dignity during the last phase of their life. However, these people were not in the last phase of their life, and we did not know whether they were able to conceive of a situation in which they were terminally ill when responding to this question. Nevertheless, the aim of this study was to determine whether the PDI can be used in people with an advance directive, because thinking in advance about dignity at the end of their life could be helpful in the organisation of advance care-planning for people who are not (terminally) ill. This study population, which consisted of people with an advance directive or a will to live statement, have probably already thought about end-of-life issues. Respondents might have thought more profoundly about end-of-life issues since they have formulated their wishes concerning end-of-life care in an advance directive which enhances the quality of the data. However, the results of this study might not be generalized to other populations since the study population consisted of two extreme groups regarding views on end-of-life care; members of the NVVE having an advance euthanasia directive, refusal of treatment statement and/or durable power of attorney, and members of the NPV, people with strong religious beliefs who declared that he/she wish for proper care, meaning no excessive, medically useless treatments at the end of life but also no actions with the purpose of actively terminating his life. Though, these two groups are very explicit and definite with regard to their views on end-of-life care issues, it is likely that the thoughts and views of the majority of the Dutch general population are covered by the results of this study.

It was noticeable that the results of this study are largely in accordance with the issues which were considered as influential to dignity in studies focusing terminally ill cancer patients by Chochinov et al. Hence, it is very likely that the findings can be generalised to populations in other countries because the explicit and definite views on end-of-life care issues also exists in other countries. For instance, 'not being able to think clearly' was found as highest ranked item in the psychological domain and 'feeling you do not have control over your life' was found as highest ranked item in the existential domain in both Chochinovs and our study[14]. However, the terminally ill cancer patients indicated more often that they (strongly) agreed that the PDI items influenced dignity. This applies, for example, to the item 'changes in physical appearance' that 66% of the terminally ill patients considered to be influential for dignity, compared to 12-18% in the present study. Therefore, it seems that some issues only become important for dignity when people are terminally ill.

### Strengths and limitations

An important strength is that this is a large-scale study. Therefore, it was possible to sub-divide the cohort into two groups, i.e. the PDI group and the group who received the open-ended question, which was important for adequate assessment of the content validity of the PDI in this study population. We assessed the content validity in a structured way, using the COSMIN checklist as a guideline for designing and reporting on the content validity of the PDI in people with an advance directive in the Netherlands.

A limitation of this study could be that the researchers who labelled the responses to the open-ended question were already familiar with the PDI. Moreover, the present study focused on the 22-item PDI prototype, and not on the final revised 25-item PDI, which was published during the period of data-collection for this study.

## Conclusion

In view of the ageing population, and the fact that people live for a longer period of their life in a poor health, understanding concerns about dignity becomes increasingly important. The present large-scale study demonstrates the relevance of the PDI items for people with an advance directive in the Netherlands. We found that, in addition to being valid for use in terminally ill cancer patients, the PDI can also be used in a general population to obtain insight into people's thoughts about what would constitute dignity in the last phase of their life. However, the comprehensiveness of the PDI items can be improved by including items concerning communication and care-related aspects. Additionally, the PDI could be improved by more specific phrasing of the items. Finally, the addition of an open-ended question to the PDI could be helpful, acknowledging the fact that what constitutes dignity is personal, and can be different for every person.

## Competing interests

The authors declare that they have no competing interests.

## Authors' contributions

All authors participated in the design and coordination of the study. GA performed the analyses. All authors conceived of the study read and approved the final manuscript.

## Supplementary Material

Additional file 1 **Table S1**. Content labels applied to the responses to the open-ended question. Click here for file
