# Supplementary material for: Analysis of the construct of dignity and content validity of the patient dignity inventory
Source: Health Qual Life Outcomes. 2011 Jun 19;9:45. doi: 10.1186/1477-7525-9-45 (PMC3141372; doi:10.1186/1477-7525-9-45)
Supplement: Additional file 1 — Table S1. Content labels applied to the responses to the open-ended question. [file 1477-7525-9-45-S1.DOC]

**Additional file 1, Table S1** Content labels applied to the responses to the open-ended question

| **Physical** | **psychological** | **social** | **existential** |
| --- | --- | --- | --- |
| **Independence** | **Mental clarity *(Not being able to think clearly)*** | ***Feeling a burden to others*** | ***Feeling you do not have control over your life*** |
| *Not being able to independently manage bodily functions* | Dementia | ***Feeling your privacy has been reduced*** | In ‘common’ life |
| Incontinence | (Sub-)comatose | Meddlesome | Unable to make decisions concerning treatment/life and death |
|  | Being a vegetable/dependent on life-support |  | No right to self-determination |
| Not being able to wash and bath independently | Disoriented in time/place | ***Not being treated with respect or understanding*** | ***No longer feeling like who you were*** |
| Not being able to eat/drink independently | Not recognising family members/loved ones | No respect for religion | ***Not feeling worthwhile or valued*** |
| *Not being able to carry out tasks of daily living* | Incompetent for decision-making | Not treated with respect | No longer feeling valuable to others |
| Immobile/bedridden | ***Feeling depressed or anxious*** | No affectionate warm/loving care | (Loss of) decorum |
| Changes in physical appearance | Fear of death | Not being taken seriously by the physician | Normal thinking/talking |
| Emaciated | Concerns about surviving relatives | Not being patronized | Self respect |
| Hair-loss | ***Not being able to accept things the way they are*** | Spiritual counselling | **Desired treatment goal** |
| Senile decay/slovenly | Problems in maintaining normal routine | Respect for life (and death) | No prolongation of life/being allowed to ’let go’ |
| Experiencing distressing symptoms | No (inner) acceptance/not at peace with oneself | Respect for the wishes of the patient/family members | No hastened death/euthanasia |
| Pain | ***Not being able to mentally fight*** | ***Not feeling supported by your community*** | Adequate pain(/symptom) management |
| Dyspnoea |  | Regarding (practical) care | Euthanasia |
| Poor eyesight |  | Regarding attention/understanding | Relief of suffering |
| Hearing problems |  | Loneliness | Palliative care |
| **Limitations in (leisure) activities** |  | Presence of loved ones/Saying good bye to loved ones | ***Feeling life no longer has meaning or purpose*** |
| *Not being able to carry out important roles* |  | **Ability to communicate** | No longer enjoying anything |
| Unable to take care of loved ones |  | As a means to indicate what you want | No prospect of improvement/incurable disease |
|  |  | As a social activity | Losing interest in people/things around you |
| *Not being able to continue with usual routines* |  |  | ***Not having a meaningful spiritual life*** |
| Hobbies |  | **Environmental aspects of care** | ***Uncertainty regarding illness*** |
| Work |  | Being cared for in a quite/safe place | ***Thinking how life might end*** |
| Not living as you want to live |  | Being cared for at home/not in an institution |  |
|  |  | Not being cared for by strangers/many different people | **OTHER** |
|  |  | Being cared for in a hospice | Adequate care/tailored care |
|  |  |  | You should not judge dignity yourself (God should do this) |

*Italic items* are PDI items
